# *Entamoeba histolytica* Trophozoites and Lipopeptidophosphoglycan Trigger Human Neutrophil Extracellular Traps

**DOI:** 10.1371/journal.pone.0158979

**Published:** 2016-07-14

**Authors:** Eva E. Ávila, Norma Salaiza, Julieta Pulido, Mayra C. Rodríguez, César Díaz-Godínez, Juan P. Laclette, Ingeborg Becker, Julio C. Carrero

**Affiliations:** 1 Department of Biology, Division of Exact and Natural Sciences, Universidad de Guanajuato, 36050, Guanajuato, México; 2 Department of Experimental Medicine, Medical Faculty, Universidad Nacional Autónoma de México, 04510, México D.F., México; 3 Department of Immunology, Instituto de Investigaciones Biomédicas, Universidad Nacional Autónoma de México, 04510, México D.F., México; The Hospital for Sick Children and The University of Toronto, CANADA

## Abstract

Neutrophil defense mechanisms include phagocytosis, degranulation and the formation of extracellular traps (NET). These networks of DNA are triggered by several immune and microbial factors, representing a defense strategy to prevent microbial spread by trapping/killing pathogens. This may be important against *Entamoeba histolytica*, since its large size hinders its phagocytosis. The aim of this study was to determine whether *E*. *histolytica* and their lipopeptidophosphoglycan (EhLPPG) induce the formation of NETs and the outcome of their interaction with the parasite. Our data show that live amoebae and EhLPPG, but not fixed trophozoites, induced NET formation in a time and dose dependent manner, starting at 5 min of co-incubation. Although immunofluorescence studies showed that the NETs contain cathelicidin LL-37 in close proximity to amoebae, the trophozoite growth was only affected when ethylene glycol tetra-acetic acid (EGTA) was present during contact with NETs, suggesting that the activity of enzymes requiring calcium, such as DNases, may be important for amoeba survival. In conclusion, *E*. *histolytica* trophozoites and EhLPPG induce *in vitro* formation of human NETs, which did not affect the parasite growth unless a chelating agent was present. These results suggest that NETs may be an important factor of the innate immune response during infection with *E*. *histolytica*.

## Introduction

Maternal and child undernutrition, highly prevalent in low- and middle-income countries, account for about 35% of deaths for children younger than 5 years[1]. The limitation of nutrients negatively impacts the immune response, predisposing to infectious diseases, among them amoebiasis and other diarrheal infections[2]. Amoebiasis caused by the protozoan parasite *Entamoeba histolytica* is ranked as the third leading parasite-associated cause of human mortality worldwide, behind malaria and schistosomiasis[3], and the second leading cause of intestinal parasitosis behind cryptosporidiosis[4]. Thus, *E*. *histolytica* was found responsible for 55,500 deaths worldwide in 2010 and it is estimated to account for 10 million cases of dysentery and liver abscesses every year. Tissue invasion of the intestine or liver by *E*. *histolytica* is associated with the induction of a strong inflammatory response characterized by the recruitment of a large number of neutrophils in the early stages[5–8]. Usually, large neutrophil infiltrates can be found surrounding trophozoites, which show no evidence of apparent damage. Therefore, the role of neutrophils in amoebiasis has always been controversial, since some groups claim that these granulocytes participate in the resolution of infection[9–14], whereas other groups suggest that they are involved in tissue damage[15–20]. However, humans with a mutation in the leptin receptor (Q223R) have increased susceptibility to amoebiasis, likely due to impaired chemotaxis and reduced gut infiltration of neutrophils, suggesting a contribution of these cells in eliminating *E*. *histolytica* in natural infection[21].

One of the innate immune mechanisms exerted by neutrophils is the formation of extracellular traps of DNA, known as NETs. NETs are complex weblike structures of decondensed chromatin decorated with granular and cytoplasmic proteins that arise from the release of the neutrophil nuclear contents under several stimulating conditions[22–25]. Among other proteins, human NETs contain cathelicidin (LL-37), a cationic antimicrobial peptide present in the specific granules and produced after the C terminal cleavage of the human cationic antimicrobial Protein 18 (hCAP-18) by serine proteases[26]. It has been described that NETs are able to trap both gram positive and negative bacteria, as well as fungi, viruses and parasites, killing or inhibiting their growth, preventing the spread of infections and thus contributing to the establishment of a protective immune response against pathogens[27]. However, conflicting reports have arisen as consequence of using different techniques to assess microbial killing, such as counting of plated colonies, where NETs are able to clump the microbes without killing them. Furthermore, the excessive development of NETs has recently begun to be associated with autoimmune and vasculitic diseases, contributing in general to the pathology of some diseases associated with microbial infections[25].

NET formation has been described to occur in response to several human protozoan parasites. Thus, these structures were identified in blood smears of children with uncomplicated malaria infected with *Plasmodium falciparum* and appeared to correlate with the presence of antinuclear antibodies, predictive of autoimmunity[28]. On the other hand, NET formation has been reported in response to *ex vivo* stimulation with *Leishmania amazonensis* amastigotes and promastigotes of *L*. *amazonensis*, *L*. *major*, *L*. *chagasi* and *L*. *donovani* promastigotes. As a result of NET-parasite interaction, *L*. *amazonensis* promastigotes were killed, whereas *L*. *donovani* survived, and *L*. *mexicana* sequestered by NETs delayed the recruitment other immune cells contributing to the persistence of skin lesions in mice[29–31]. NETs are also triggered with *Toxoplasma gondii* infections, killing approximately 25% of the entangled parasites, which suggests a protective role to contain the infection[32]. Recently, an *in vitro* induction of NETs, dependent on the signaling through toll-like receptors (TLRs), was reported for *Trypanosoma cruzi*[33]. Although the NETs were unable to kill the parasite, they did decrease the number of infected cells and the number of released trypomastigote forms. Taken together, the role of NETs in parasitic infections remains unclear and further studies are warranted. In this context, the role of NETs in the viability of *E*. *histolytica* and the pathogenesis of amoebiasis has not been characterized.

## Results

### *E*. *histolytica* trophozoites trigger NET formation in human neutrophils

**Fig 1:**
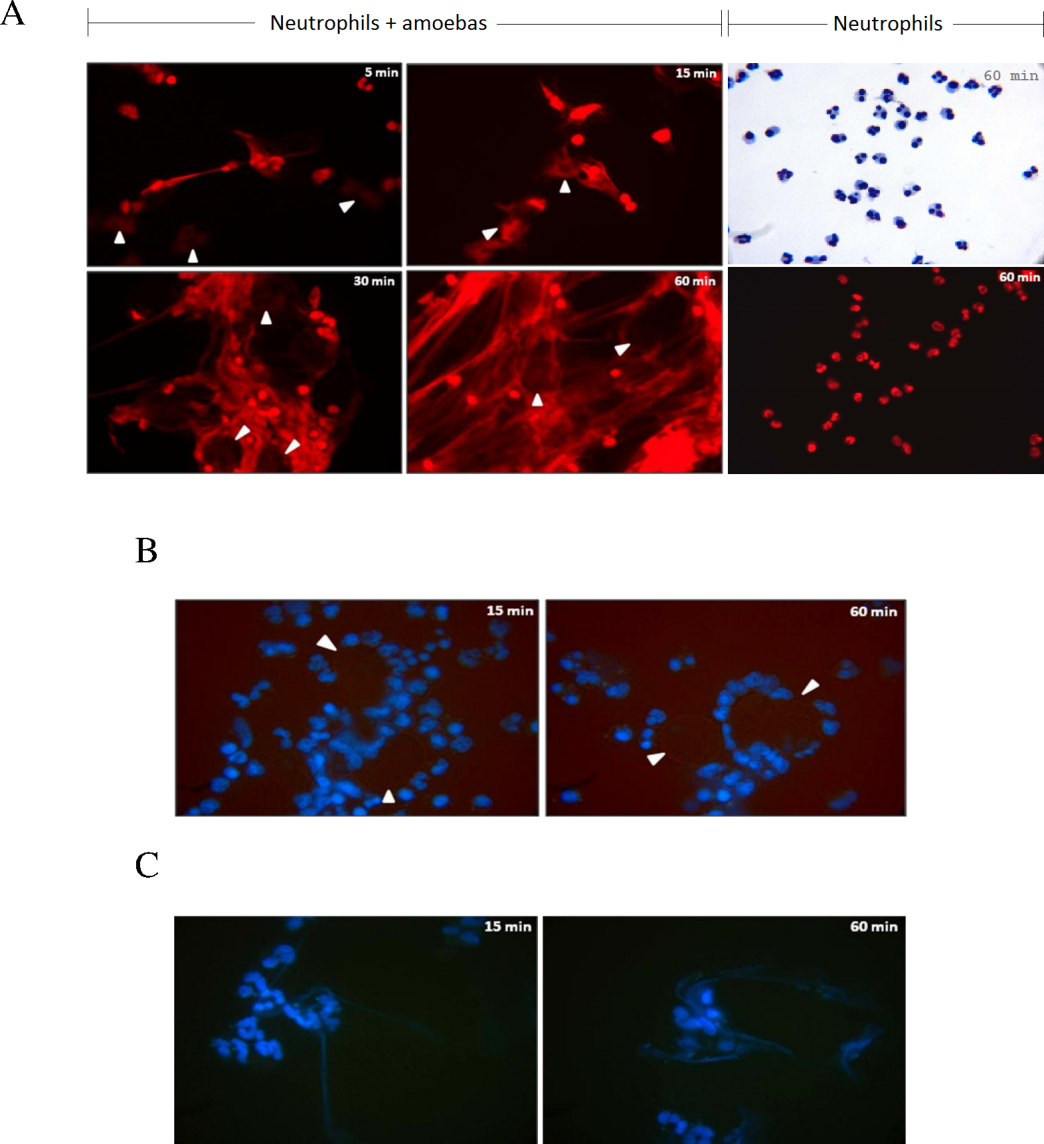
*E. histolytica* trophozoites induce the formation of human neutrophil extracellular traps. A) Human neutrophils isolated by positive selection from peripheral heparinized blood were incubated with *E*. *histolytica* HM1:IMSS trophozoites (ratio 1 amoeba to 20 neutrophils) and the release of NETs monitored with 7AAD stain at 5, 15, 30 and 60 mins. Spider web-like fibers are observed as rapid as 5 min of incubation and increase in number and density over time. The networks were initially seen projected out of neutrophils toward amoebas (5 and 15 mins), and later gradually increase in number until completely cover the trophozoites that seem to be snared in the mesh (30 and 60 mins). Neutrophils in the absence of amoebas and incubated for 60 min are shown stained with Giemsa or 7ADD.B) Incubation of isolated human neutrophils with formaldehyde-fixed trophozoites did not induce NETs at 15 and 60 min. In A and B, trophozoites location is indicated by white head-arrows; magnification 40X. C) Incubation of isolated human neutrophils with fresh trophozoites whole extract induces scarce NETs at 15 and 60 min. B and C were stained with Hoechst.

Incubation of *Entamoeba histolytica* with human neutrophils (ratio 1:20) trigger their rapid ejection of 7-aminoactinomycin D (7AAD)-stained thin filaments being ejected from neutrophils. These fibers were clearly observed beginning after 5 min of incubation and increasing in number and density over time, showing a tangle of chunky meshworks that cover almost the entire field of vision at 60 min post-incubation (Fig 1A). Release of DNA was not observed in unstimulated neutrophils within the hour of incubation (Fig 1A). Furthermore, amoeba-induced NET formation was also dose-dependent, since the increase of the trophozoite:neutrophil ratio (1:10) resulted in higher number of NET formations (data not shown). NET networks were seen in close contact with trophozoites, surrounding them after 30 min, suggesting that trophozoites were trapped in these structures (Fig 1A, see arrows at 30 and 60 min). However, amoebic trophozoites showed no apparent morphological or size changes within the 60 min of their interaction, and additionally retained their ability to phagocytose PMNs (S1 Fig).

To analyze the role of NETs on trophozoite viability and integrity in NET formation, human neutrophils were incubated at the same ratio with previously paraformaldehyde-fixed trophozoites or with fresh whole extracts. It is noteworthy that fixed trophozoites did not trigger NET formation during the hour of their co-incubation, despite that most of the neutrophils interacted with the surface of the fixed trophozoites (Fig 1B). Whole extracts from trophozoites at 1 mg/mL triggered scarce NET formation that increased slightly over time (Fig 1C). These results indicate that *E*. *histolytica* induced NET formation seems dependent on the trophozoite integrity.

### Human NETs are unable to kill *E*. *histolytica* trophozoites

**Fig 2:**
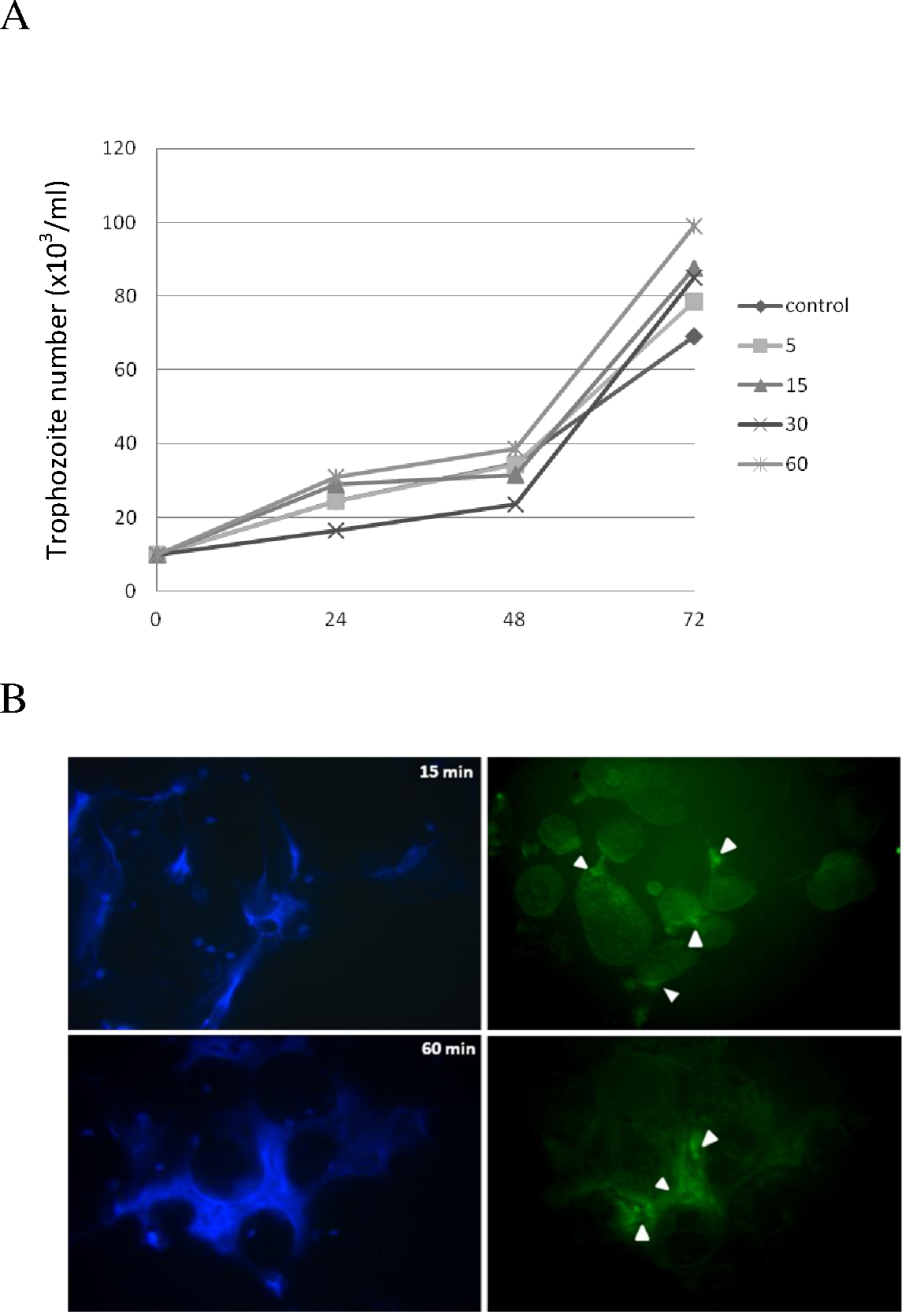
Trophozoites induced NETs are unable to inhibit parasite growth despite containing anti-microbial cathelicidin LL-37. A) Growth kinetic of trophozoites co-incubated with NETs for the indicated times (5 to 60 min) and thereafter cultured in fresh TYI-S-33 medium. Live trophozoites were counted every 24 h under light microscope using Trypan blue. Data shown at each time is the mean ± SD of three independent assays. B) Immunofluorescence assay upon NETs induced by amoebas at 15 and 60 min (left panels) using anti-LL-37 antibody and an anti-rabbit IgG conjugated to FITC (right panels). Magnification 40X.

In order to analyze the role of human NETs on the viability of *E*. *histolytica*, trophozoites were co-incubated with human neutrophils (ratio 1:20) for 5, 15, 30 and 60 min, washed and cultured for additional 72 h in fresh TYI-S-33 medium. Counting of viable trophozoites every 24 h showed that the NET formation from human neutrophils did not affect the viability and growth of amoebas at any of the co-incubation times tested (Fig 2A). A slight decrease was observed in the growth curves of trophozoites after 24 and 48 h of their exposure to NETs for 30 min, but the differences were not statistically significant. In fact, trophozoites exposed to NETs tended to grow better than control trophozoites at 72 h, albeit the differences were not statistically significant (Fig 2A).

Since cathelicidin LL-37 has previously been shown to affect the integrity of *E*. *histolytica* trophozoites[34], we analyzed whether cathelicidin LL-37 formed part of the antimicrobial peptide (AMP) in the NETs induced by *E*. *histolytica* trophozoites. The immunofluorescence results show that the NETs induced by *E*. *histolytica* contain cathelicidin LL-37, clearly visible at sites of interaction between neutrophils and amoebas as early as 15 min after the co-incubation, which increases over time. After 60 min, a network tangle containing cathelicidin LL-37 was found surrounding the trophozoites (Fig 2B, white arrows; Upper and lower panels). These results suggest that *E*. *histolytica* trophozoites could be resistant to the cathelicidin LL-37 and other antimicrobial peptides found within human NETs induced *in vitro* by the parasite.

**Fig 3:**
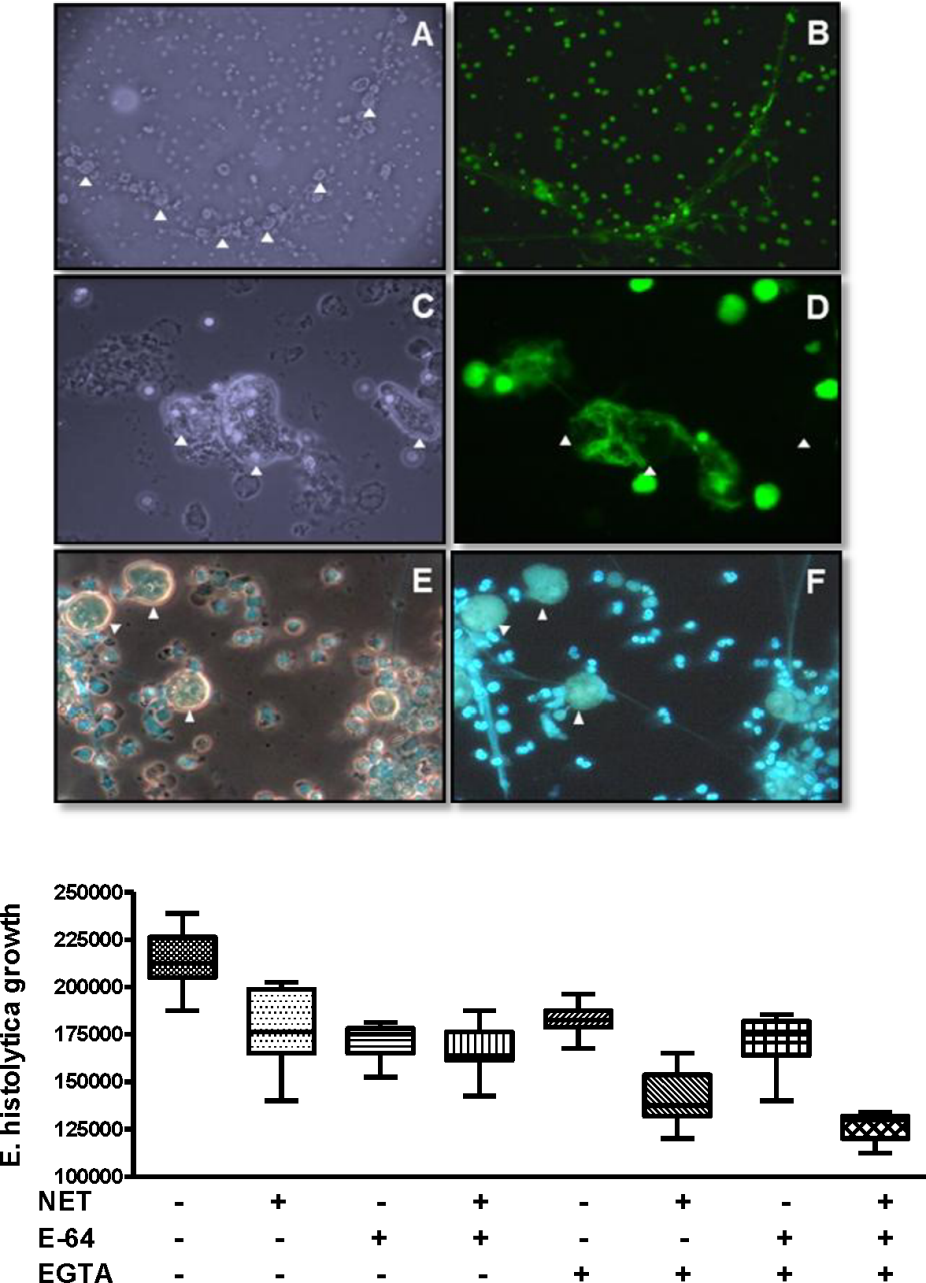
Interaction and growth of *E*. *histolytica* trophozoites with neutrophil extracellular traps. NETs induced with PMA were incubated 1 h with amoeba trophozoites. Top: A-D, Sytox Green; E and F, Hoechst 33342. A, B at 10X and C-F at 40X objectives at the fluorescence microscope. A and C, phase contrast fields corresponding to B and D, respectively. F, florescence; E, merges phase contrast with fluorescence. Arrow tips, *E*. *histolytica* trophozoites. Bottom: after amoeba-NET interaction, trophozoites were growth in TYI-S-33 culture medium during 72 h and cells harvested were counted under a light microscope. Controls, trophozoites cultured at the same conditions in the absence of NETs. Amoeba growth after interaction with NETs and EGTA or EGTA + E-64 was significantly different from controls without NETs (p <0.001). No other significant difference (p <0.05) was observed compared to the control without NETs in the absence of other compounds.

Considering the possibility that the NET formed in the presence of *E*. *histolytica* trophozoites lacked other AMPs or other granule-derived anti-microbial molecules present in typical drug-induced NETs, we evaluated the effect of human NETs, formed from human neutrophils previously induced with phorbol myristate acetate (PMA), on the amoeba viability. As shown in Fig 3, most of the amoebae were in close contact with the fibers of DNA, either as rosary beads along the fibers (panels A and B), entirely surrounded by the networks (panels C and D) or interconnected through spider web-like structures (panels E and F). Trophozoites were polymorphic and were negative for Sytox Green staining, a DNA dye that does not permeate live cells, suggesting that most of the trophozoites were viable at 1 h of exposure to PMA-induced NETs. To confirm their viability, these trophozoites were washed and cultured in fresh TYI-S-33 medium for additional 72 h. The count of viable *E*. *histolytica* trophozoites showed that trophozoites exposed to PMA-induced human NETs grew less as compared to non-treated amoebas, however, the difference was not statistically significant (Fig 3, bottom graph). In order to determine the possible mechanism of resistance of trophozoites to human NETs, the effect of the protease inhibitor E64 as well as the chelator ethylene glycol tetra-acetic acid (EGTA) was analyzed on the viability of amoeba exposed to PMA-induced NETs. Both E64 and EGTA were used in order to inhibit possible *E*. *histolytica* secreted/excreted cysteine proteases and DNases, respectively, which could degrade AMPs or DNA that constitute NET. The results show that E-64 did not affect the growth of trophozoites within NETs, which was similar to controls. In contrast, the addition of EGTA, a chelator of divalent ions, had a deleterious effect on the growth of amoeba within the NET, alone or combined with E-64 (p<0.001; Fig 3, bottom graphic). This result suggests that a trophozoite-associated DNase activity may be responsible for the resistance of *E*. *histolytica* to the deleterious effect of human NETs.

### *E*. *histolytica* LPPG can trigger NET formation in human neutrophils

**Fig 4:**
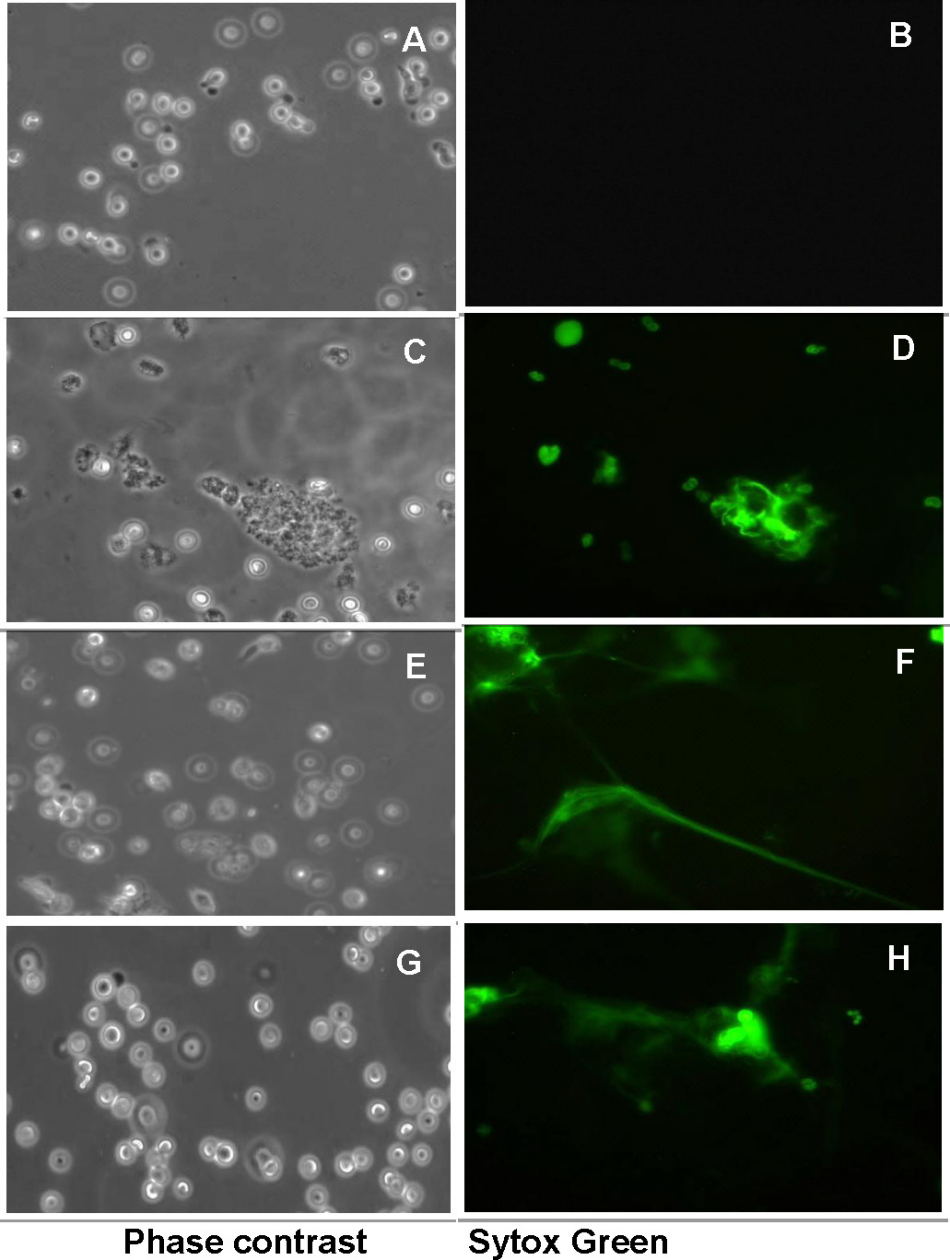
Induction of neutrophil extracellular traps by different concentrations of lipopeptidophosphoglycan from *E*. *histolytica*. Human neutrophils were incubated for 2 h at 37°C with 0 to 15 ng/μL of purified EhLPPG. A, C, E and G, phase contrast fields of B, D, F, and H, respectively. Sytox Green observed at 40X objective in the fluorescence microscope.

In order to identify some of the molecules of amoeba involved in the formation of NETs, we evaluated whether purified *E*. *histolytica* lipopeptidophosphoglycan (EhLPPG) triggered NET formation in human neutrophils. Results show that isolated EhLPPG at concentrations ranging from 5 to 15 ng/μL induces the formation of NETs in a dose-dependent manner (Fig 4, panels D, F, H). Non-treated neutrophils did not change their morphology and did not expulse DNA, remaining non-stained with Sytox Green during the 2 h incubation (Fig 4, panel B), indicating a specific induction of NETs by EhLPPG.

**Fig 5:**
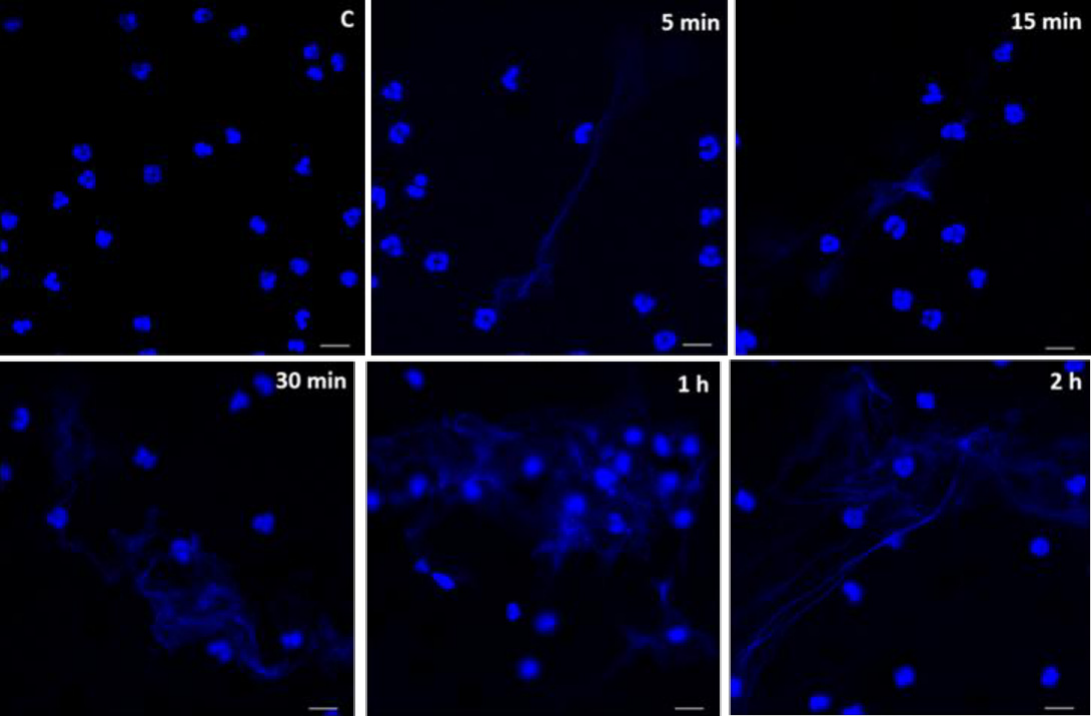
Kinetics of NET induction by lipopeptidophosphoglycan isolated from *E*. *histolytica*. Induction of neutrophil extracellular traps by 10 ng/μL of EhLPPG during the times indicated. Control, neutrophils incubated at the same conditions for 2 h in the absence of EhLPPG. Hoechst 33342 stain and confocal microscope observation, bars 10 μm.

It is noteworthy that EhLPPG-induced NET formation initiates as early as 5 min after exposure and increases over time (Fig 5), similarly as observed for trophozoites. At 5 and 15 min of exposure, most NETs consisted of thin DNA fibers, while after 30 min NETs were observed as more complex DNA networks. Untreated neutrophils remained without morphological changes throughout the experiment (panel C).

**Fig 6:**
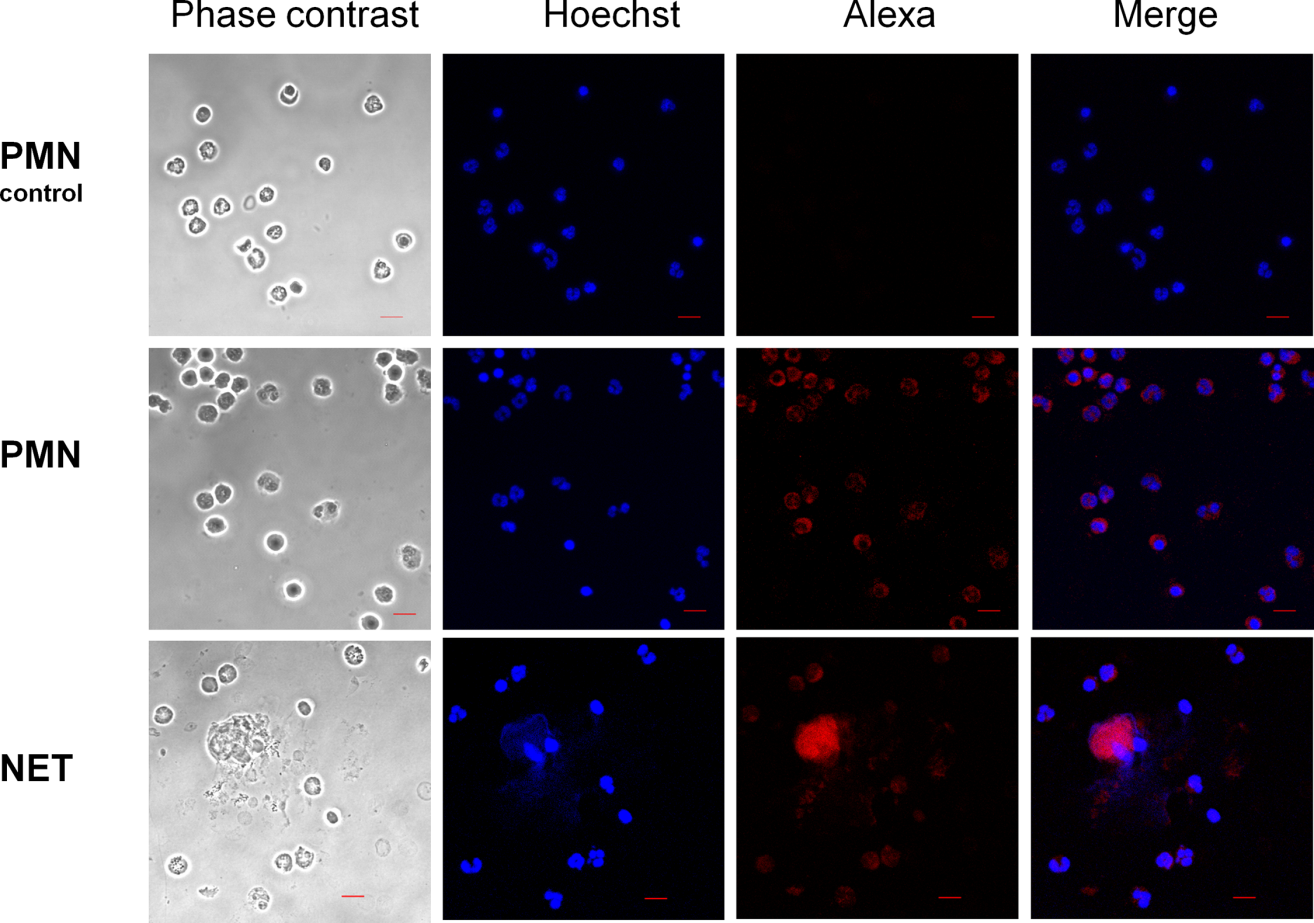
Localization of LL-37 in NET induced by LPPG from *E*. *histolytica*. Neutrophils or NET induced by EhLPPG were stained by an anti-LL-37 antibody and an anti-rabbit IgG conjugated to Alexa594 (red). Control PMN, polymorphonuclear leucocytes with only the secondary antibody. PMN, PMN stained with anti-LL-37 and the anti-rabbit IgG conjugated to Alexa594. NET, PMN incubated with LPPG showing the release of NET and colocalization with LL-37. Hoechst 33342 stain and confocal microscope observation, bars 10 μm.

In addition, immunofluorescence analysis show that neutrophil´s cathelicidin LL-37 (Fig 6E and 6F) co-localize with the NETs induced by EhLPPG (Fig 6H and 6I), suggesting that this granule AMP is released bound to the neutrophil DNA ejected upon stimulus with the surface component of amoeba.

### PMA-preformed NETs did not affect the infectivity of *E*. *histolytica*

**Fig 7:**
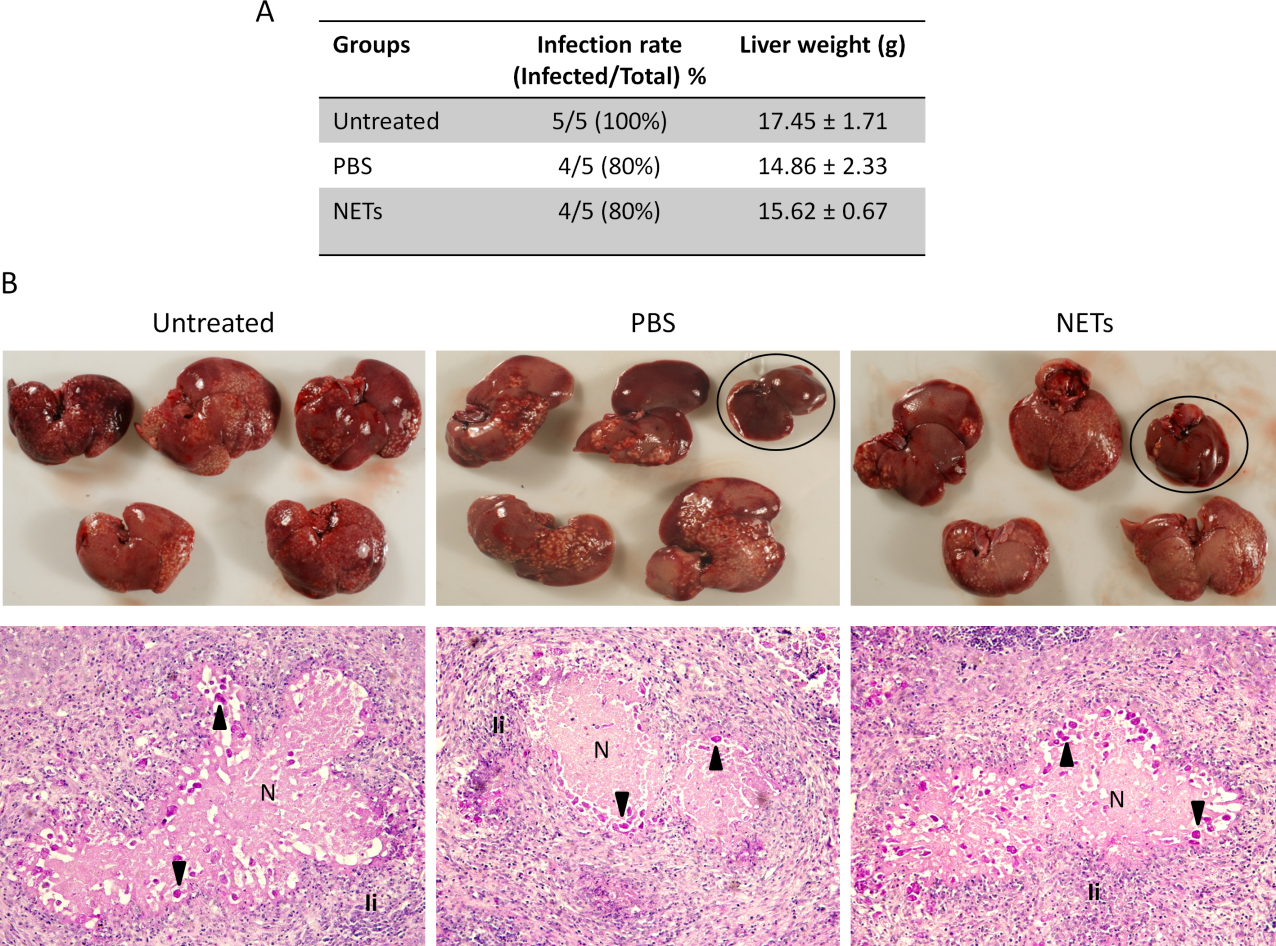
Development of amoebic liver abscesses (ALA) on hamsters by amoebas pre-treated with NETs. Virulent *E*. *histolytica* trophozoited (1x10^6^) were incubated with 500 μg DNA NET solution (PMA-preformed) or PBS for 1 h at 37°C, and then injected in the portal vein of hamsters. Untreated trophozoites were used as control of infectivity. Seven days post-challenge, animals were sacrificed, livers escinded and analyzed. A) Table showing the infection rate and weight average of infected livers. B) Upper: Distribution and magnitud of ALA on the liver of the 5 animals of each group. Uninfected livers of groups PBS and NETs are inside ovals. Bottom: Representative histological analysis of ALA from each group stained with PAS. Magnification 20X. Arrows: trophozoites; N: necrosis; Ii: Inflammatory infiltrate, mainly neutrophils.

In order to assess whether NETs exert any effect on the infectivity of *E*. *histolytica*, we determined the ability of trophozoites pretreated for 1 h with PMA-induced NETs to develop amoebic liver abscesses (ALA) in hamsters, when compared with trophozoites treated with PBS or untreated. Massive development of ALA throughout the liver was observed in all 5 animals of the untreatred group and 4 out 5 animals of the PBS and NETs-treated groups (Fig 7A and 7B). Although one animal did not develop ALA in the group pretreated with NETs, this effect appears to be due to the incubation of amoeba with PBS for 1 h, since one animal of that group did not either develop ALA. The hepatomegaly mesured as the average weight of livers, an indirect indication of ALA extent, was also similar between groups when compared only animals that developed ALA (Fig 7A). A comparative macroscopic description of infected livers showed similar grades of ALA development in the three groups, with many abscessses of variable size distributed in all lobes (Fig 7B). Similar results were also observed when ALA were analyzed in histological sections. Thus, large abscesses formed by coalescence of many small were observed throughout the liver tissue, containing well-preserved amebas close to the edges of lesions, abundant PMN cell infiltration and necrosis (Fig 7C). No clear differences in the the extent of injuries, population of immune cells recruited to the ALA and number and integrity of amoebas were observed between groups. Overall our results suggest that *in vitro* obtained human NETs did not affect the pathogenicity of *E*. *histolytica* evaluated in the model of ALA in hamsters.

## Discussion

Neutrophils are the first polymorphonuclear cells recruited to infection sites and are among the first line of defense of the cellular innate immunity[35]. Over a decade ago, networks of extracellular DNA containing granule proteins and histones, known as NETs, were described. NET formation can occur under multiple stimuli including reactive oxygen species[36], antibodies and antigen–antibody complexes[37,38], TLR4-activated platelets[39] and microbes such as many bacteria and some virus, fungi and protozoa[40]. NET formation in response to protozoan parasites has been reported for *L*. *amazonensis* amastigotes and *L*. *amazonensis*, *L*. *major*, *L*. *chagasi*, *L*. *donovani* and *L*. *mexicana* promastigotes[29–31], *Toxoplasma gondii* tachyzoites[32], *Trypanosoma cruzi* trypomastigotes[33] and *Eimeria arloingi* sporozoites and oocysts [41]. However, characterization of NETs induced by the protozoan parasite *E*. *histolytica* causing amoebiasis in humans has not been previously described. We here demonstrate that human neutrophils release NETs upon exposure to amoebic trophozoites as well as to its surface molecule EhLPPG. In accordance with other reports on NET formation in the presence of microbes, the trophozoites of *E*. *histolytica* trigger NET formation rapidly (starting at 5 min) in a dose and time dependent manner. This effect seemed dependent on amoeba viability, since fixed trophozoites were unable to elicit them. It is noteworthy however that slight and retarded NET formation was induced by total *E*. *histolytica* extracts as well as by conditioned medium (not shown), suggesting that microbial components present in both preparations can also trigger NET formation.

Among the microbial molecular triggers of NETs are the LPS of gram negative bacteria[23] and the LPG of *Leishmania amazonensis* promastigotes[29], the most prominent negatively charged surface components involved in the activation of the host innate immune response trough TLR 2 and TLR 4[42,43]. In this study, we showed that purified EhLPPG, a surface molecule structurally similar to Leishmania LPG, was also able to elicit NET formation in a similar manner to viable trophozoites, suggesting that EhLPPG is one of the main parasite molecules triggering traps in human neutrophils. This GPI-anchored proteophosphoglycan[44] is a highly immunogenic molecule directly exposed to the host’s immune system, recognized by the sera from patients with amoebic liver abscess[45]. The mechanism by which EhLPPG is able to activate the release of NETs was not addressed in this manuscript and remains unknown. However, since it was described that EhLPPG is a molecular pattern that triggers the host immune response by signaling through TLR2/TLR4[43], it is conceivable that those pattern recognition receptors and the same pathway are responsible for the NET release by EhLPPG. Interestingly, other studies using genetically engineered *L*. *donovani* showed that NET induction by promastigotes is independent of the parasite surface LPG, suggesting that other parasite molecules are responsible for NET triggering in these species[30]. Studies are underway in our laboratory in order to identify other molecules, in addition to the EhLPPG, that are possibly involved in NET formation.

Amoebic viability assays by growth in fresh medium demonstrated that *E*. *histolytica* trophozoites were not significantly affected after co-incubation with the NETs, induced by the amoeba itself or by classical stimuli such as PMA. In some cases, trophozoites grew equally well or even better than untreated cultures, indicating that at least under the conditions tested *in vitro*, amoebas were able to evade the microbicidal effect of human NETs. Similar findings of resistance to NETs were seen in viability or infectivity studies of other parasites such as *L*. *major*[29], *L*. *donovani*[30], and *L*. *mexicana*[31], as well as in the infectivity on *T*. *cruzi*[33]. In the latter case, although the NET did not cause the parasite death, it interfered with the ability of *T*. *cruzi* to infect LLC-MK2 cells, suggesting a protective role independent of a direct NET trypanocide effect. A similar effect of human NETs on the infectivity of *E*. *histolytica* cannot be ruled out and is currently being studied by our group. One possible explanation for the inability of amoeba-induced NETs to kill the trophozoites *in vitro* was attributed to the fact that these NETs were atypical and thereafter possibly lacked anti-microbial molecules derived from the granules of neutrophils, including cathelicidin LL-37, an AMP that had previously been shown to decrease the growth of *E*. *histolytica* trophozoites[34]. Yet this possibility was ruled out since immunofluorescence studies using an antibody against LL-37 showed that this AMP was present in the *E*. *histolytica*- and EhLPPG-induced NETs, and increased with time of exposure. Since treated trophozoites grew well in culture medium we speculate that *E*. *histolytica* possesses evasion mechanisms for NETs, which was also observed when the amoebas were exposed to NETs obtained through other stimuli, such as PMA.

Our study additionally showed that the viability of amoeba showed a slight but significant decrease when treated with EGTA, a chelator used to inhibit some enzymes including DNases. In contrast, the viability was not affected after incubation with E-64, a potent cysteine protease inhibitor, suggesting *E*. *histolytica* possibly secreted/excreted DNases could be involved in the evasion mechanisms of amoeba against human NETs *in vitro*. The role of DNases in protection against NETs has been reported for many bacteria such as group A *Streptococcus*[46], *Staphylococcus aureus*[47,48], *Streptococcus agalactiae*[49], *S*. *pneumoniae*[50], *Vibrio cholera*[51], *S*. *sanguinis*[52], *Neisseria gonorrhoeae*[53] and *S*. *suis*[54]. Recently, a 3’-nucleotidase/nuclease enzyme that allows *Leishmania infantum* to survive after interaction with NET was reported[55]. In this regard, a restriction enzyme-like endonuclease activity was shown in *E*. *histolytica* trophozoites[56], yet it is not clear whether this or any other DNase that degrades NETs is secreted by the parasite, allowing it to escape and survive.

In addition to the killing of pathogens, NETs are able to trap microorganisms and to degrade virulence factors[23]. We show here that amoeba trophozoites are sequestered by NETs *in vitro*; if this occur in the gut infection, parasite elimination may be easier by peristalsis, decreasing amoebic colonization. Another possible effect of traps on trophozoites is the degradation of amoeba virulence factors. On the other hand, as NET formation involves oxygen reactive species, the excessive formation of tramps by neutrophils may also contribute to tissue injury. In this regard, studies have shown the inability of amoeba to induce amoebic liver abscesses in neutropenic animals by treatment with immunosuppressors[20, 57]. These results together with our observation in this study showing the inability of NETs to inhibit *in vitro* viability and the infectivity of the amoeba in the ALA model in hamsters, suggests that if NETs are formed *in vivo* during infection with the amoeba, they could be contributing to tissue damage more than to protection against the parasite. Further studies are required to address these points.

In conclusion, our study shows that both *E*. *histolytica* trophozoites as well as the isolated lipopeptidophosphoglycan (EhLPPG) triggers NET formation by human neutrophils in a dose- and time-dependent manner. Furthermore, we show the presence of the granule protein cathelicidin LL-37 among the components of the DNA trap, yet it appears not affect the viability of *E*. *histolytica* trophozoites. Therefore, this study suggests that human NET formation participates in the early innate immune response against *E*. *histolytica*. However, it remains to be established whether NETs protect against natural amoebiasis *in vivo*, and if they contribute to the tissue damage associated with the infection.

## Material and Methods

### Ethics Statement

The study was reviewed and approved by the Ethics and Research Committees of the Faculty of Medicine of UNAM (Universidad Nacional Autonoma de Mexico) (FMED/CI/RGG/ 013/01/2008) and guidelines established by the Mexican Health Authorities were strictly followed. All patients and controls were informed and signed a written consent to participate in the study.

### Culture of *Entamoeba histolytica* trophozoites

*E*. *histolytica* trophozoites strain HM1: IMSS were cultured axenically at 37°C in screw-capped glass tubes containing TYI-S-33 medium[58], supplemented with 15% heat-inactivated bovine serum, 100 U/mL penicillin, 100 μg/mL streptomycin sulfate, and 1.5% Diamond vitamin mix. At the end of their logarithmic growth phase (72 h), the trophozoites were chilled on ice for 5 min and harvested by centrifugation at 150 ×*g* for 7 min at 4°C, washed three times with PBS, and suspended in RPMI-1640 with 2% human serum albumin (RPMI-HSA).

### Lipopeptidophosphoglycan isolation from *Entamoeba histolytica* trophozoites

Lipopeptidophosphoglycan was isolated from *Entamoeba histolytica* trophozoites (EhLPPG) as reported elsewhere[59]. All the glassware was heated at 250°C for one hour before use; sterile plastic material and pyrogen free water were used throughout the process. At the end of purification, EhLPPG was concentrated by lyophilization and total carbohydrates were determined by Fenol-H_2_SO_4_ method[60]. EhLPP was analyzed[61] in 12% SDS-PAGE and gels stained with Coomassie blue (negative stain), silver stain and periodic acid Schiff (PAS) reagent (not shown). The potential contamination of EhLPPG with bacterial lipopolysaccharide was ruled out by the analysis of chromogenic limulus amebocyte lysate (Lonza Mexico, catalog 27A-50-647U), according to the supplier's instructions. The LPS content in EhLPPG preparations used at 10 ng/μL was at or below 0.07 endotoxin units/mL.

### Human neutrophil isolation

Neutrophils were isolated from peripheral heparinized blood, obtained from human healthy volunteer donors using Histopaque 1119 and 1077 (Sigma-Aldrich), according to the manufacturer instructions. The granulocyte layer was collected and after washing with cold PBS, erythrocytes were lysed by incubation in lysis buffer (155 mM NH4Cl, 10 mM KHCO3, 0.1 mM EDTA) for 10 min on ice, followed by washings with cold PBS and centrifugation at 300 x*g* for 10 min at 4°C. Neutrophils were highly purified from this preparation by positive selection. Briefly, 5x10^7^ cells were suspended in 50 μl of cold PBS and 50 μl of CD16 micro beads (Miltenyi, Biotec, Bergisch Gladbach, Germany), incubated for 30 min at 4°C, washed with PBS, centrifuged at 300 x*g* for 10 min and passed through a magnetic separation LS column (Miltenyi Biotec, Bergisch Gladbach, Germany). Finally, purified human neutrophils were placed in RPMI-1640 (Gibco) with 2% human serum albumin (CSL Behring) and seeded at 1-2x10^5^ cells on sterile 8-well Cover Chamber Slides (Thermo), and incubated at 37°C under 5% CO_2_ atmosphere during 1 or 2 h with the different stimuli to induce NET formation as mentioned below.

### Induction of neutrophil extracellular traps

NET formation was induced with live or 4% paraformaldehyde fixed *E*. *histolytica* trophozoites (ratios 1:20 and 1:10 of amoeba:neutrophil), 1μg of trophozoites fresh whole extract/1 x 10^5^ neutrophils or 5, 10 and 15 ng/μL of lipopeptidophosphoglycan isolated from *E*. *histolytica* trophozoites (EhLPPG). Culture plates were incubated at 37°C under 5% CO_2_ atmosphere for variable times, beginning at 5 min to a maximum of 2 h, as indicated on each experiment. For NET staining and microscopy observation, samples were fixed with 2 to 4% paraformaldehyde (Sigma-Aldrich) for 15 min, air dried and stained with 50 μg/mL 7-ADD, 10 μg/mL Hoechst 33342 (Invitrogen) or 0.2 μM Sytox Green (Life Technologies). The coverslips were mounted with Prolong Gold (Invitrogen) before observation at the confocal (Carl Zeiss, LSM700) or fluorescence (Carl Zeiss, AxiosKop40) microscopes.

### Detection of cathelicidin LL-37 in neutrophil extracellular traps

For detection of LL-37, the neutrophils were seeded and NETs were induced as stated above, with the exception that coverslips were treated with poly-L-lysine before use. Samples were fixed with 4% paraformaldehyde or acetone for 10 min, air dried and washed with Tris HCl 0.1 M pH 7.4. Unspecific protein binding was blocked with a solution of 10% human plasma, 0.1% gelatin and 0.1% Tween 20 in PBS for 1 h at room temperature. The slides were incubated with a polyclonal anti-human cathelicidin LL-37 antibody (Santa Cruz Biotechnology) at 1:200 dilution over night at 4°C, washed with Tris-HCl buffer and incubated with a secondary antibody for 1 h at room temperature, either 1:250 diluted goat anti-rabbit IgG conjugated to Alexa594 (Life technologies) or 1:100 diluted goat anti-rabbit IgG conjugated to biotin (Zymed). In the last case, Steptavidin AP 1:100 (KPL) was added and incubated for 30 min, washed and developed with AP RED Kit (Zymed), counterstained with hematoxylin and mounted with Aqua Monter (Bio SB). Controls without first antibody were included and nuclei were stained with Hoechst 33342. All the samples with fluorescence and immunocytochemistry were analyzed with a Carl Zeiss microscope (Axio Imager M1) and the microphotographs were taken with the AxioCam MRc5 digital camera (Carl Zeiss).

### Interaction of *E*. *histolytica* with PMA-preformed neutrophil extracellular traps

NET formation was induced for 2 h with 25 nM PMA as described elsewhere[23]. A total number of 20,000 or 40,000 trophozoites per well were added to achieve a ratio of one amoeba to 20 neutrophils (1:20). Final volume was 1 mL of RPMI-HSA with or without cysteine-HCl (1 g/L), ascorbic acid (0.2 g/L) and ferric ammonium citrate (0.0236 g/L) at pH 7.0. These compounds were used in some experiments at the final concentrations found in the culture media of Entamoeba, in order to maintain trophozoites in good conditions and assure that any change in amoeba viability was due to the presence of NETs. Some experiments were performed in the presence of 0.5 mM EGTA, 10 μM E-64 or both to inhibit *Entamoeba histolytica* enzymes potentially present (proteases and DNases). Appropriate controls were included such as seeding the same number of trophozoites in wells without NETs. Plates were incubated for 1 h at 37°C under a microaerophilic atmosphere (most of the oxygen was consumed by a burning candle in a closed anaerobic jar), and samples were treated for microscopic observation or to determine the amoeba growth.

### *E*. *histolytica* growth assays

Trophozoites that induced NETs at 5, 15, 30 or 60 min, as well as trophozoites exposed to PMA-preformed NETs during 2 h, were harvested and transferred to screw-capped tubes with 5 mL TYI-S-33 medium, and cultured for 72 h in habitual culture conditions for amoeba. Trophozoites were harvested at the indicated times and counted in a Neubauer chamber using the vital stain Trypan blue. Data were analyzed by Kruskal-Wallis test, considered statistically significant a p<0.05.

### Induction of amoebic liver abscesses in hamsters

*E*. *histolytica* trophozoites from 72 h cultures, harvested by ice-chilling during 5 min and washed three times with PBS pH 7.2, were treated with NETs obtained by treatment of human neutrophils with 25 nM PMA for 2 h as described above. Briefly, batches of 1x10^6^ parasites were incubated with 500 μg DNA NET solution or PBS for 1 h at 37°C. Each million of treated trophozoites were collected by centrifugation at 150 ×*g* for 7 min at 4°C, washed three times with PBS, suspended in 100 μl PBS and used for infection of hamsters as described before[20]. Male Syrian golden hamsters (Mesocricetus auratus) 4 to 6 weeks of age were maintained free of pathogens with water and food *ad libitum*. Following a protocol approved by the Institutional Animal Care Committee, animals were divided in 2 groups of 5 hamsters each (Untreated and treated groups). The animals were anaesthetized with anesthesal (60 mg⁄ kg) and the portal vein was exposed by laparatomy under aseptic conditions. Untreated or NETs-treated trophozoites (1x10^6^/hamster) processed as above were directly inoculated into the portal vein using a tuberculin syringe, followed by the immediate application of a gel-foam pad at the site of inoculation in order to avoid bleeding and loss of amoebas.

### Histological analysis

Hamsters were sacrificed after 7 days and the livers removed and weighed. Abscess were exscinded from the liver tissue and also weighed. Liver samples containing abscesses were fixed in 4% paraformaldehyde in PBS for 1 h, embedded in paraffin and processed for histology by standard techniques. In brief, serial sections of 20 μm thicknesses were obtained in a microtome, placed on slides coated with poly-L-lysine (Sigma, St Louis, MO, USA), deparaffined, and finally stained with haematoxylin/eosin or Periodic acid–Schiff for light microscopy analysis.

## Supporting Information

S1 Fig Phagocytosis of human neutrophils by NETs-treated *E*. *histolytica* trophozoites. Trophozoites were exposed for 1 h to PMA-preformed NETs (25 nM) and then incubated with neutrophils previously fixed with paraformaldehyde 4% and stained with DAPI for 10 min (A). Aliquots were taken at 15 min (B) and 30 min (C) and observed in a fluorescence microscope. Upper: light microscopy; middle: UV microscopy; bottom: simultaneous light and UV microscopy. All images were taken at 40X.
(TIF)
