# Supplementary figures and images for: Entamoeba histolytica Trophozoites and Lipopeptidophosphoglycan Trigger Human Neutrophil Extracellular Traps
Source: PLoS One. 2016 Jul 14;11(7):e0158979. doi: 10.1371/journal.pone.0158979 (PMC4944907; doi:10.1371/journal.pone.0158979)

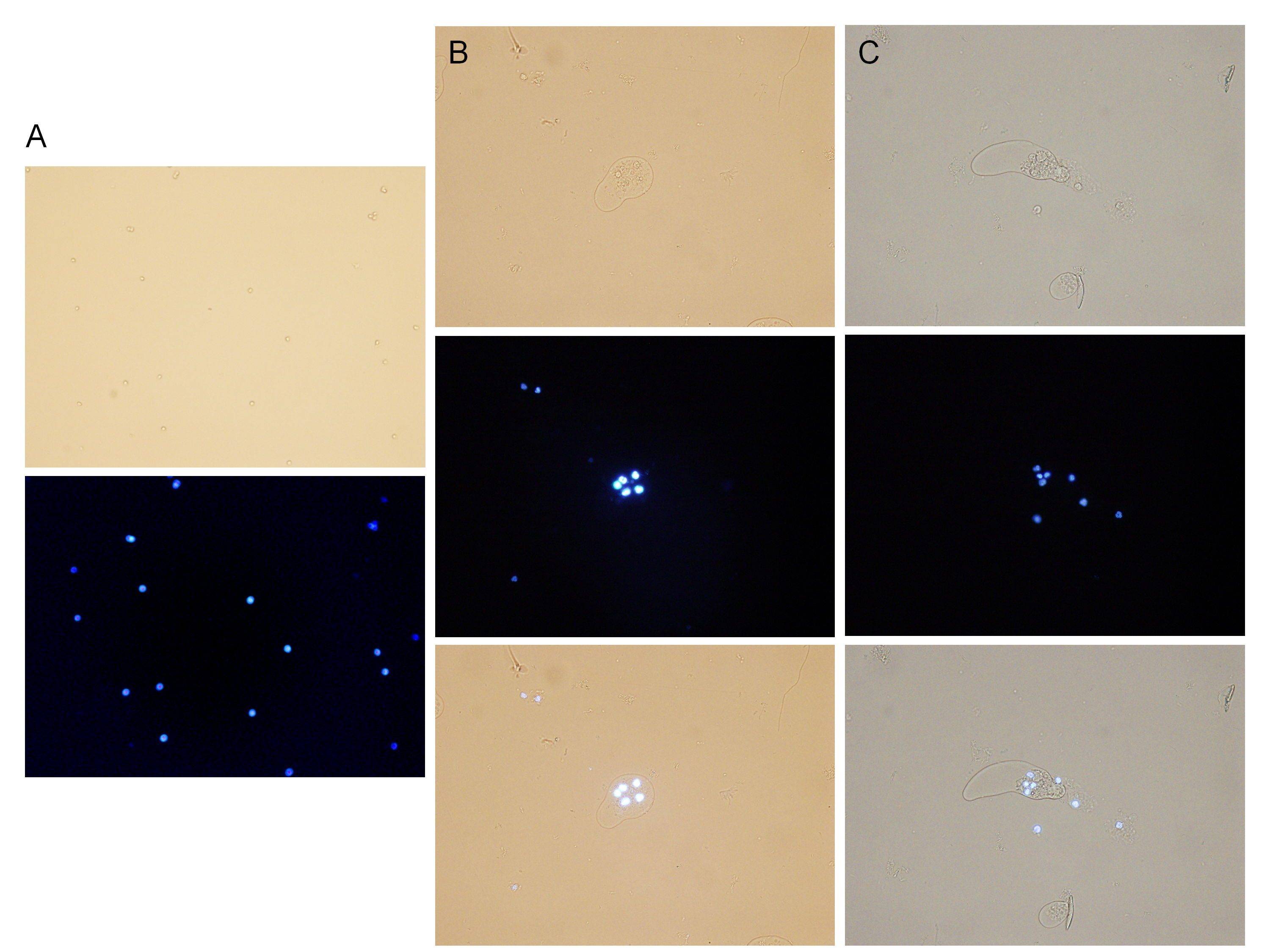

Supplement: S1 Fig — Trophozoites were exposed for 1 h to PMA-preformed NETs (25 nM) and then incubated with neutrophils previously fixed with paraformaldehyde 4% and stained with DAPI for 10 min (A). Aliquots were taken at 15 min (B) and 30 min (C) and observed in a fluorescence microscope. Upper: light microscopy; middle: UV microscopy; bottom: simultaneous light and UV microscopy. All images were taken at 40X. (TIF) [file pone.0158979.s001.tif]
